# Examining Voting Capacity in Older Adults with and without Cognitive Decline †

**DOI:** 10.3390/brainsci12121614

**Published:** 2022-11-25

**Authors:** Eleni Poptsi, Despina Moraitou, Marianna Tsatali, Vasileios Papaliagkas, Maria Tzanakaki-Melissari, Elia Kyriakoulaki, Fotini Kounti, Nefeli Markou, Despina Liapi, Georgia Batsila, Fani Ouzouni, Maria Vasiloglou, Magda Tsolaki

**Affiliations:** 1 Laboratory of Psychology, Department of Cognitive and Experimental Psychology, Faculty of Philosophy, School of Psychology, Aristotle University of Thessaloniki (AUTh), 54124 Thessaloniki, Greece; 2 Lab of Neurodegenerative Diseases, Center for Interdisciplinary Research and Innovation, Aristotle University of Thessaloniki (CIRI—AUTh), 54124 Thessaloniki, Greece; 3 Greek Association of Alzheimer’s Disease and Related Disorders (GAADRD), 54643 Thessaloniki, Greece; 4 Department of Psychology, School of Humanities and Social Sciences, University of Western Macedonia, 50100 Kozani, Greece; 5 Department of Biomedical Sciences, International Hellenic University, 57001 Thessaloniki, Greece; 6 Institute of Research and Education of Psychiatric and Dementia Patient’s, 73100 Chania, Greece; 7 Heraklion Association of Alzheimer’s Disease and Healthy Aging “ALLILENGII”, 71201 Heraklion, Greece; 8 1st Department of Neurology, Medical School, Aristotle University of Thessaloniki (AUTh), 54124 Thessaloniki, Greece

**Keywords:** voting capacity, general cognitive ability, dementia, Alzheimer’s Disease, cognitively healthy status

## Abstract

Background: Nowadays, controversy exists regarding the stage of cognitive decline and/or dementia where voting capacity is diminished. Aim: To evaluate whether general cognitive status in advancing age predicts voting capacity in its specific aspects. Methods: The study sample comprised 391 people: 88 cognitively healthy older adults (CH), 150 people with Mild Cognitive Impairment (MCI), and 153 people with Alzheimer’s disease dementia (ADD). The assessment included CAT-V for the voting capacity and Mini Mental State Examination (MMSE) for general cognitive ability. ANOVAs and ROC curves were the tools of statistical analysis towards (a) indicating under which MMSE rate participants are incapable of voting and (b) whether the CAT-V total score can discriminate people with dementia (PwADD) from people without dementia (PwtD). Results: Out of the six CAT-V questions, one question was associated with a low MMSE cutoff score (19.50), having excellent sensitivity (92.5%) and specificity (77.20%), whilst the other five questions presented a higher MMSE cutoff score, with a good sensitivity (78.4% to 87.6%) and specificity (75.3% to 81.7%), indicating that voting difficulties are associated with cognitive status. Secondarily, the total CAT-V score discriminates PwADD from PwtD of 51–65 years (sensitivity 93.2%/specificity 100%—excellent), PwADD from PwtD of 66–75 years (sensitivity 73.3%/specificity 97.1%—good), PwADD from PwtD of 76–85 years (sensitivity 92.2%/specificity 64.7%—good), whilst for 86–95 years, a cutoff of 9.5 resulted in perfect sensitivity and specificity (100%). Conclusion: According to MMSE, PwADD have no full voting competence, whilst PwtD seem to have intact voting capacity. The calculated cut-off scores indicate that only people who score more than 28 points on the MMSE have voting capacity.

## 1. Introduction

In democratic countries, voting is a fundamental legal right. However, people who are denied access to elections exist, such as people with cognitive and emotional disorders [1]. Nowadays, a subject of controversy concerns the voting capacity of people with cognitive impairment, discussing whether they should remain active voters [2].

Alzheimer’s disease dementia (ADD), as well as Mild Cognitive Impairment (MCI), are characterized by decline in a broad range of cognitive domains [3,4,5,6,7,8,9]. Decision making, as a complex process that generates and evaluates choice alternatives in older people [10] and as a part of the executive function spectrum, is also impaired in people with dementia due to Alzheimer’s disease (PwAD) [11]. On the contrary, people with MCI (PwMCI) seem to have a relatively preserved abstract thinking ability and the capacity for reasoning [12]. Therefore, people with Alzheimer’s disease dementia (PwADD) potentially face problems with the voting process, in contrast to people with MCI. Nevertheless, the data providing evidence regarding the lack or the maintenance of voting capacity in MCI are scarce in the literature.

As far as ADD is concerned, even though no international guidelines exist regarding whether PwADD are capable to vote, a 2001 federal district court decision proposed that people are considered incompetent to vote only if “they lack the capacity to understand the nature and the effect of voting in such a way that they cannot make an individual choice” (Doe, 2001, pp. 14–15) [13]. However, the stage of ADD where people lose the skills related to voting is a topic of controversy, since if one exists it will provide a quantitative metric via which people can lose their rights to vote, creating interference with their legal rights, and therefore raising political issues. Prior research about the voting capacity of PwADD has shown that a dementia diagnosis does not automatically presuppose a lack of voting capacity, since the latter seems to be related to several factors, one of which being the degree of cognitive impairment [14,15].

Based on the above, some interesting questions emerge. First, whether PwADD are capable of participating in the voting process, and to be more precise, to what extent PwADD are capable of understanding, making a choice, performing reasoning, and appreciating the voting process. This question is crucial, since the world’s population is rapidly aging, and as a consequence the prevalence of dementia among older adults is increasing [16]. Furthermore, statistics indicate that participation rates in the voting process are higher for older people (including people with very mild, moderate or even severe dementia) when compared to the respective younger rates [17]. Another emerging question, and probably of the utmost importance nowadays, is how to objectively evaluate voting capacity. People with cognitive impairment exist (e.g., PwADD) who, even though they desire to vote and are capable of voting, are denied their right due to their condition. On the other hand, there are people who have lost voting capacity but are still active voters due to lack of recent cognitive assessment [18].

In order to assess the competence to vote, Appelbaum, Bonnie and Karlawish in 2005 [15] designed the Competence Assessment Tool for Voting (CAT-V). The CAT-V is based on standard decision-making abilities such as understanding, choice, reasoning, and appreciation. According to Grisso and Appelbaum in 1998, understanding is the ability to comprehend the meaning of information and the benefits and harms of the options, while appreciation is defined as the ability to recognize how the information applies to a person and comprehend the risks versus benefits of the possible solutions range [19]. On the other hand, “*Reasoning ability refers to the power and effectiveness of the processes and strategies used in drawing inferences, reaching conclusions, arriving at solutions, and making decisions based on available evidence*” [20]. Solving complex reasoning problems seems to depend on executive control abilities, and specifically on the capacity to maintain and manipulate information in working memory [21]. Decision-making abilities, including the aforementioned, seem to play a crucial role in voting capacity, yet they are impaired even in people with mild neurocognitive disorders [22,23]. Decision making is considered closely related to the ability of choice. “*Choice is the outcome of a process which involves assessment and judgement; that is, the evaluation of different options and making a decision about which option to choose*” [24]. The ability to choose, as well as decision-making ability, seem to be affected by the emotional status or/and cognitive deficits noticed in neurodegenerative diseases such as Alzheimer’s disease. Therefore, since voting capacity is related to abilities that are usually impaired in people with dementia (decision making and choice), it is hypothesized that these people may have difficulties in understanding or participating in the voting process.

Until now, only a few studies exist that have evaluated the voting capacity of people diagnosed with cognitive impairment and dementia. Specifically, the study by Appelbaum, Bonnie, and Karlawish, who designed the CAT-V test [15], indicates that performance on the CAT-V’s questions is strongly correlated with Mini Mental State Examination (MMSE). Furthermore, patients with very mild to moderate ADD retain adequate ability to vote, whilst people with severe ADD do not. Additionally, it was noticed that the desire to vote was independent of voting capacity. Tiraboschi, Chito, Sacco, Sala, Stefanini, and Defanti (2011) [25] also found that voting ability was correlated with MMSE as well as with the ADD stage. Moreover, according to their results, MMSE does not predict voting capacity, whilst CAT-V does. Therefore, it seems that ADD patients had a lower performance in the questions of CAT-V related to understanding, reasoning and decision making, besides the fact that the question related to choice making seems to be the least affected by dementia [25]. Finally, Irastorza, Corujo, and Banuelos (2011) [14] noticed a correlation between MMSE and CAT-V, since people without cognitive impairment seemed to have a greater capacity to vote, associating the MMSE score with voting capacity. Besides the fact that people with mild ADD seemed to retain the capacity to vote, a great variability existed as far as moderate ADD was concerned. Therefore, the authors concluded that CAT-V is a valid tool for severe ADD, but not for mild and moderate ADD [14].

Voting capacity in people with cognitive disorders is not adequately studied worldwide, even though it poses a fundamental human right issue and obligation of citizens. Even though a few relatively recent studies exist evaluating the capacity to vote in the PwADD population by using the CAT-V, as well as its correlation with MMSE, their number is quite small and they display several limitations regarding the size of the sample, the sample’s randomization, or the lack of control group, e.g., cognitively healthy older adults [15,25]. Moreover, in Greece, similarly to several European countries, no studies exist investigating the voting capacity of healthy older adults and older adults with minor and major cognitive disorders, such as PwMCI and dementia. Therefore, it is essential to investigate at which stage of cognitive decline and/or dementia people lose their decision-making and reasoning abilities, using cognitive assessment tools such as the CAT-V. Such an investigation would be beneficial for policy makers or/and scientists, since it could lead to new voting guidelines established for PwADD. It would also be useful for caregivers with difficulty to realize whether their patients can make the right choice or have the ability of reasoning, in order to assist them with participating in the voting process in case the patients want to.

## 2. Purpose and Hypotheses of the Study

Since no normative data for the Greek older adult population exist, the main aim of the present study was to assess whether and to what extent the general cognitive status of older adults (measured via MMSE) can predict specific aspects of voting capacity. On the contrary, we were also interested in examining if general voting capacity (via CAT-V) can discriminate PwADD from PwtD.

The hypotheses were formulated as follows:

**Hypothesis** **1.** *The higher the total MMSE score, the higher the voting capacity categorization of the participant, derived from CAT-V, assuming existence of complex cognitive abilities in voting.*

**Hypothesis** **2.** *The higher the total CAT-V score, the healthier the cognitive status of older adults will be.*

## 3. Methods

### 3.1. Design

In the current study, 391 older adults participated, categorized into three different cognitive stages (CH, PwMCI, PwADD). The study sample consisted of the visitors of (a) the 2 Day Care Centres of the Greek Association of Alzheimer’s Disease and Related Disorders’ “Saint Helen” and “Saint John” in Thessaloniki, and (b) the Institute of Research and Education of Psychiatric and Dementia patients, in Chania, Crete, from 2015 to 2017. As far as healthy older adults were concerned, they visited the Day Care Centres for their yearly neuropsychological and psychological checkup routines, at which time they were asked if they desired to take part in the “capacity to vote” study. For every CH older adult who agreed to participate in the study, a new appointment in the Day Care Centres or in the Institute of Research in Crete was set about 5 to 10 days after their initial neuropsychological assessment. All PwMCI due to AD and PwADD were participating in the training cognitive programs of the Day Care Centres and the Institute of Research in Crete during the same period. The training process in the aforementioned centers included training of attention and memory abilities, which are not associated with executive function abilities, such as abstract thinking, choice, and reasoning. In this way, the training programs could not affect the participants’ performance in the CAT-V test, constituting a convenient sample with an already complete extended neuropsychological battery administered by trained neuropsychologists. The CAT-V was administered to all participants of this study within 5 to 10 days after the administration of MMSE, as a part of their neuropsychological examination, and thus reliability issues due to different administration timing were avoided.

Finally, because of the study’s nature and the time required for the CAT-V implementation (approximately 5 min), there were no missing data and therefore no need for extra samples.

### 3.2. Ethical Standards

All the participants (healthy older adults, MCI and PwADD) gave written informed consent at the time of their initial clinical visit, agreeing that the trained research staff of the Greek Association of Alzheimer’s Disease and Related Disorders and the Institute of Research in Crete could use their basic demographic information such as age, gender, and education, as well as their total scores of neuropsychological tests, for research reasons. They also gave written informed consent for participating in the CAT-V study at the time of the initial neuropsychological assessment (5 to 10 days prior), and they were aware that they could withdraw their consent at any time without their statutory rights or medical care being affected. For PwADD, a legal representative provided the informed consent, them being incapable of providing it themselves. The study was approved by the Scientific and Ethics Committee of the Greek Association of Alzheimer’s Disease and Related Disorders (identifying number 161/2015) which follows the new General Data Protection Regulation (EU) 2016/679 of the European Parliament and of the Council of 27 April 2016 on the protection of natural persons with regard to the processing of personal data and on the free movement of such data, as well as the principles outlined in the Helsinki Declaration.

### 3.3. Participants

The study sample included 391 Greek native speakers who underwent the CAT-V from 2015 to 2017. The sample was comprised of 105 men and 286 women, with mean age *M* = 72.04, *SD* = 8.69 (ranging from 51–95 years) and mean years of education *M* = 10.98, *SD* = 4.71 (ranging from 0–23 years) (Table 1). At this point it should be mentioned that the participants belonged to three diagnostic groups: (a) Cognitively Healthy Older Adults, (*n* = 88, 18 men and 70 women, with mean age *M* = 66.08, *SD* = 7.93 years, and mean education *M* = 13.43, *SD* = 3.78), (b) PwMCI due to AD according to DSM-5 [6], (*n* = 150, 30 men and 120 women, with mean age *M* = 70.47, *SD* = 7.98 years, and mean education *M* = 11.77, *SD* = 4.58), and (c) PwADD according to DSM-5 [6], (*n* = 153, 57 men and 96 women, with mean age *M* = 77.01, *SD* = 6.90 years, and mean education *M* = 8.86, *SD* = 4.34). According to their MMSE scores [26], the ADD group contained 42 people with very mild ADD, (27.5%), 43 people with mild ADD (28.1%), 36 people with moderate ADD (23.5%), and 32 people with severe ADD (20.9%). The severity of ADD was evaluated using the standard cut-off points of the MMSE: very mild dementia: 24–30 points; mild dementia: 20–23 points; moderate dementia: 12–19, severe dementia: less than 12 [26]. The diagnosis was supported by a consensus of specialized neurologists and neuropsychologists, after neurological examination and neuropsychological assessment, medical history, neuroimaging (computed tomography or magnetic resonance imaging), and blood tests. The 3 groups (CH, PwMCI, PwADD) differed in age, *F*(3390) = 53.44, *p* < 0.001, gender, χ^2^(2391) = 13.84, *p* < 0.001 and educational level according to the years of education, χ^2^(6390) = 48.41, *p* < 0.001. In this study, all participants were treated as a single group of older adults experiencing different rates of cognitive status.

The exclusion criteria for all participants comprised (a) affective disorders such as severe depression, assessed by the Geriatric Depression Scale (GDS) [27,28] in participants over 65 years and Beck Depression Inventory (BDI) [29] for participants < 64 years, or anxiety, assessed by the Short *Anxiety Screening Test* (SAST) [30,31]; (b) behavioral problems such as aggressiveness and irritability, assessed by the Neuropsychiatric Inventory (NPI) [32,33]; (c) brain and neurological diseases, such as brain tumor, encephalitis, epilepsy, stroke history, Parkinson’s disease, or hydrocephalus; (d) general severe health problems such as cancer in the last 5 years, myocardial infarction in the last 6 months, pacemaker and bypass; (e) thyroid and diabetes; (f) history or existence of any psychiatric illness, such as major depression, schizophrenia, substance abuse, alcoholism, or any other psychiatric illness that led to hospitalization; (g) history of traumatic brain injury; (h) drug treatment with opioids, benzodiazepines, depression medications, antipsychotics. The medical and psychiatric disorders were excluded using an extensive medical history, the accusative blood test, and neuroimaging such as the *Magnetic resonance imaging* (*MRI*). Participants were excluded from the study because of the aforementioned criteria since they could affect both the diagnosis validity and the CAT-V performance as well.

The inclusion criteria of the study were (a) an age larger than 50 years old and (b) a diagnosis of minor and major neurocognitive disorders according to DSM-5 criteria [6], for PwMCI and PwADD participants, respectively.

### 3.4. Measures

#### 3.4.1. The CAT-V Assessment

The CAT-V was used after written permission from the publishing author [15]. For the needs of the study, a Greek adaptation of the CAT-V test was prepared. For developing the Greek version of the questionnaire, the steps of the procedure that the World Health Organization proposes were used: (a) forward translation by qualified translators; (b) problematic terms and phrases were highlighted by the translators; (c) back-translation by independent linguists other than the original translators; (d) pre-testing and cognitive interviewing; and (e) the final version (https://tinyurl.com/253fptkd, accessed on 3 January 2011). Because the voting systems of Greece and the USA (where the test was initially designed) are quite different, the CAT-V was adjusted in order to better represent the Greek voting system.

The CAT-V competence is a tool designed by Appelbaum et al. in 2005 in order to assess the voting ability of people with dementia. The tool’s developer was based on the Doe voting capacity standard [13] for determining the voting competence. The Doe voting capacity standard includes the understanding of the nature and the effect of voting, as well as the ability to choose a candidate [15]. Afterwards, the constructor, based on the above criteria, designed a brief questionnaire adding inquiries of appreciation and reasoning about voting choices [15]. The CAT-V interview lasted approximately 5 min and included 6 questions adjusted to the Greek election system. As far as the adaptation is concerned, the only part of the questionnaire that was crucial to undergo alteration was the replacement of the word “candidates” with the phrase “political parties”, as well as the word “governor” with the word “government”, since the voters in Greece decide between “political parties”, and not between “candidates”, in order to vote for “government” and not for “governor”. The rest of the items and the questions remained the same as the prototype. The questions were the following: whether the examinee (a) understands the nature of the voting (what Greek people will do to select the next government); (b) understands the results/effects of the voting (when the election process is finished, how it will be decided who the winner is); (c) can make a choice (imagine the following situation: political party A thinks that… and political party B says that… Which political party do you think you are most likely to vote for, A or B?); (d) can compare two political parties and explain their choice (how is voting for [subject’s choice] better than voting for [name of other political party]?); (e) can identify the effect of the examinee’s choice of political party over their own life (“If [subject’s choice, or political party A if subject had no choice] were elected government in your country, how could that affect your life?”); and (f) would like to vote in the country’s next elections and why (would you want to vote in the next election for government of your country? If yes, why? If no, why not?).

According to the test constructors, the scoring of each item is 2 points if the performance is adequate, 1 point if the performance is doubtful, and 0 points if the person is not capable of processing or answering the question. For example, regarding the question of understanding the results/effects of voting, the instructors propose that the examinee is graded (a) with a score of 2 when there is a completely correct response, e.g., “The votes will be counted and the person with more votes will be the winner”, (b) with a score of 1 if there is an ambiguous or partially correct response, e.g., “By the numbers”, and (c) with a score of 0 when the response is incorrect or irrelevant, e.g., “It all depends on which Zodiac sign they were born under” [15]. Therefore, the lowest CAT-V sum score that indicates a total loss of voting ability is 0, whilst the best score is 12 (six items scored with 2 each), indicating perfect voting capacity. As far as the interpretation of the answers, two researchers of the study were responsible for independently scoring the patients’ answers. The two researchers had to completely agree regarding their categorization in all 6 questions. In the opposite case, a third researcher was responsible for resolving disagreement for the questions that had been differently classified.

It should be stated that the psychometric properties of the CAT-V assessment for the Greek population were not evaluated in the current work; nevertheless, this is one of the next targets of our research team. The version of the CAT-V assessment in Greek is presented in the included annex.

#### 3.4.2. Mini-Mental State Examination (MMSE)

The Mini-Mental State Examination is the most well-known and commonly used short and reliable screening psychometric tool for the assessment of general cognitive performance. It assesses orientation in time and place, memory, attention, naming, comprehension, execution of oral and written instructions, and written language. The best score is 30, indicating excellent cognitive performance, whilst the minimum score is 0. According to its standardization in the Greek population, a score of 23–24 was found to be the cut-off point for the differentiation between people with and without dementia. Finally, according to the Greek validation study, the MMSE is influenced by age and education [26,34].

### 3.5. Data Statistical Analysis

The statistical analysis was performed with IBM SPSS Statistics for Windows, Version 23.0 [35].

**Abbreviations: CAT-V**: Competence Assessment Tool for Voting.

As regards the total score of the CAT-V, which can be considered as a quantitative value (in terms of the interpretation of the higher score as higher general voting capacity), we proceeded with Analysis of Variance (ANOVA) applied for assessing (a) the effects of individual demographic factors (age group, educational level, gender) and (b) MMSE-related categories of cognitive status on the total score of the CAT-V. Specifically, we decided to classify the sample in two separate categories according to MMSE cut-off in Greece [27], the first concerning people with Alzheimer’s dementia disease ‘PwADD’ (MMSE ≤ 23), and the second people without dementia ‘PwtD’ (MMSE ≥ 24). At this point, it is worth mentioning that the MMSE was adopted as a measure of general cognitive status since it has already been used as a criterion of cognitive status from all cross-cultural studies regarding CAT-V. Nowadays, it is well-known that the MMSE can effectively discriminate between dementia and healthy older adults, but is not able to diagnose other milder disorders, such as Mild Cognitive Impairment (MCI), as well as Subjective Cognitive Decline (SCD). Therefore, people with the initial diagnosis of MCI and with a MMSE ≥ 24 were categorized in the PwtD group according to their exact MMSE score. The PwMCI group was included in the PwtD, since besides the fact that these are people with higher possibility of conversion to dementia, they do not fulfill the criterion of dysfunctionality and therefore they are more similar to the HC group. Nevertheless, at this point it must be mentioned that in the present study and as regards the main aim of the study, only the exact MMSE score was used to examine the specific aspects of voting capacity of the participants.

Finally, the area under the curve (AUC) of the ROC curve was used in order to quantify the CAT-V’s total score ability to discriminate cognitive decline and cognitive health, as represented by MMSE’s categories for separate age groups, into fair, good, perfect, or excellent, according to the relative literature (an AUC value of 1.0 is perfect, 0.9–0.99 is excellent, 0.8–0.89 is good, 0.7–0.79 is fair and 0.51–0.69 is denoted as poor [36,37].

## 4. Results

The answer types in each question of the CAT-V were categorical (adequate, doubtful, inability/none). Taking into account that there were a few answers classified as doubtful and none, the Receiver Operating Characteristic curve (ROC curve) analysis was used for assessing the ability of the MMSE total score to discriminate between adequate ability and inability of voting. The frequencies of the values “doubtful” and “none” as well as the frequencies of “adequate” voting are presented in Table 2.

### 4.1. Ability of the MMSE Exact Score to Discriminate Adequate Voting Capacity from Inability as Regards Specific Aspects/Categories—Questions of the CAT-V

**Figure 1:**
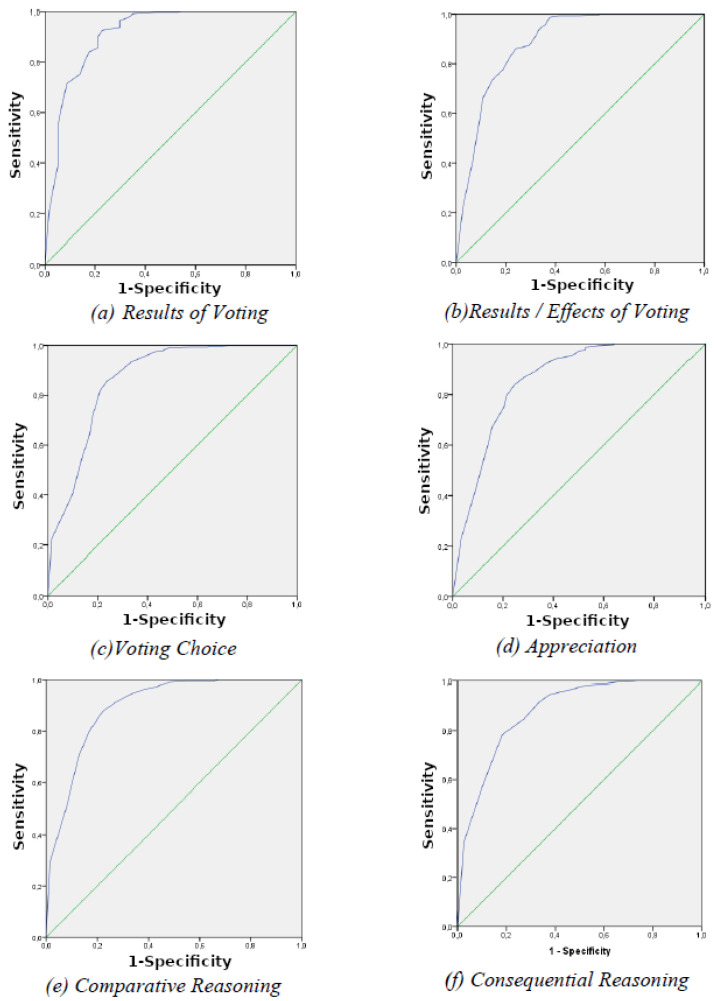
ROC curve analyses of the six sub-questions: (**a**) nature of voting, (**b**) results/effects of voting, (**c**) voting choice, (**d**) appreciation, (**e**) comparative reasoning, (**f**) consequential reasoning.

Each voting question was separately analyzed in order to assess in which out of the six questions participants showed cognitive difficulties. In other words, we attempted to investigate from which MMSE score and above the participants were capable of voting. ROC curve analysis was applied, indicating that there are some questions requiring a low score in MMSE, whilst others require a higher one. The results of the ROC curves are presented in Figure 1 below, depending on the MMSE score, sorted from lower to higher scores.

As far as the first two questions of the CAT-V were concerned, the ROC curve of the nature of voting question showed that a cutoff of 19.50 at MMSE resulted in excellent sensitivity (92.5%) and specificity (77.20%) (AUC = 0.91 95% CI = 0.86–0.96, *p* < 0.001), whilst regarding the results/effects of voting, the ROC curve showed that a cutoff of 22.50 resulted in good sensitivity (86.1%) and specificity (76%) (AUC = 0.88 95% CI = 0.83–0.93, *p* < 0.001). As far as the voting choice was concerned, the results indicate that a cutoff of 22.50 also resulted in good sensitivity (85.6%) and specificity (76.4%) (AUC = 0.86 95% CI = 0.81–0.92, *p* < 0.001). Regarding appreciation, a cutoff of 23.50 resulted in good sensitivity (84.1%) and specificity (75.3%) (AUC = 0.85 95% CI = 0.80–0.90, *p* < 0.001), whereas for comparative reasoning, a cutoff of 25.50 resulted in good sensitivity (87.6%) and specificity (78%) (AUC = 0.89 95% CI = 0.86–0.92, *p* < 0.001). Finally, as far as the consequential reasoning (consequences of voting) was concerned, the results showed that a cutoff of 27.50 resulted in good sensitivity (78.4%) and specificity (81.7%) (AUC = 0.87 95% CI = 0.84–0.91, *p* < 0.001).

### 4.2. Demographic Effects and Effects of General Cognitive Status on the Total Voting Score of CAT-V

At the next step, we proceeded to assess whether demographics (age-group, educational level, gender) and the MMSE score (as two categories of cognitive status) affected the voting capacity of the CAT-V’s total score. As far as age was concerned, the sample was categorized into four subgroups: (a) middle-aged adults (51–65 years), (b) young-old adults (66–75 years), (c) old adults (76–85 years), and (d) oldest-old adults (86–95), whereas as far as the educational level was concerned, the sample was categorized into three levels according to the number of years of schooling: (a) 0–6 years (low), (b) 7–12 years (middle), and (c) ≥13 years (high) of schooling (see Table 1). Furthermore, two separate categories regarding MMSE were created, the first concerning people with Alzheimer’s disease dementia (PwADD) (MMSE ≤ 23) and the second including people without dementia (PwtD) (MMSE ≥ 24). ANOVA was applied for assessing the effects of age group, educational level, gender, and MMSE (as categories of cognitive status) on the total score of the CAT-V. ANOVA was used in order to identify the factors that affect the performance in CAT-V (not only the factors concerning general cognitive function but also the demographic factors, which are potentially correlated variables based on the characteristics of the diagnostic groups that comprised the sample).

ANOVA results indicated that there was a significant main effect of age group, *F*(3391) = 3.16, *p* < 0.05, and a significant main effect of MMSE categories, *F*(1391) = 130.4, *p* < 0.001, on the total performance in CAT-V. Moreover, a significant interaction effect of age group and MMSE categories, *F*(3, 391) = 4.60, *p* < 0.05, was noticed. The mean scores of CAT-V according to general cognitive status and age group are presented in Table 3.

### 4.3. Ability of the CAT-V Total Score to Discriminate PwADD from PwtD in Each Age Group

**Figure 2:**
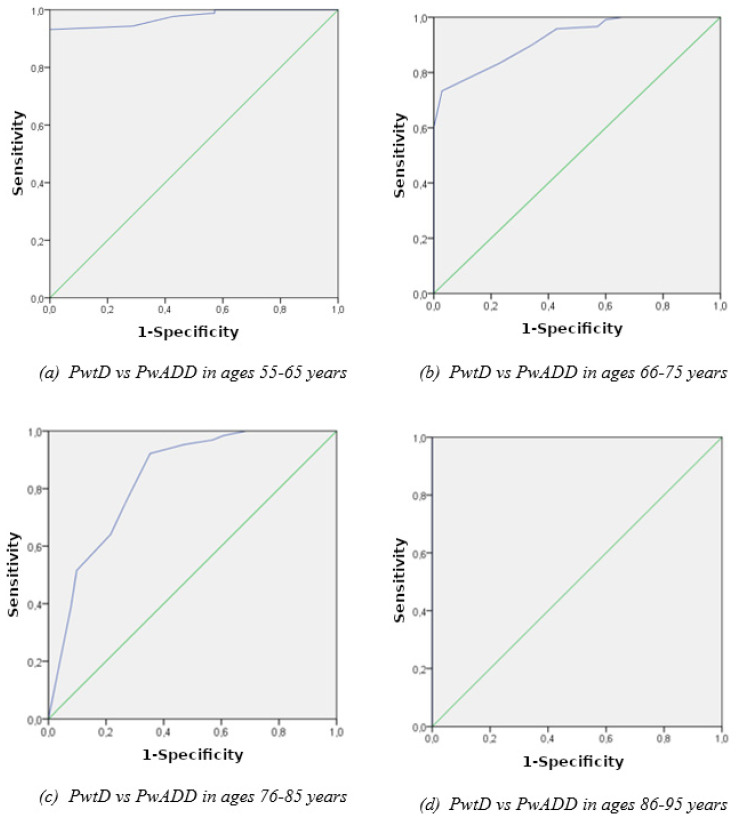
ROC curve analyses of the CAT-V total score of groups: (**a**) Group 1 (55–65 years of age), (**b**) Group 2 (66–75 years of age), (**c**) Group 3 (76–85 years of age), (**d**) Group 4 (86–95 years of age).

Based on the results of the ANOVA, ROC curve analysis for each age group was performed in order to examine whether the CAT-V total score can detect cognitive status in each age group. According to the results in middle-aged adults, CAT-V had an excellent sensitivity and specificity to discriminate PwADD from PwtD, whilst in the young-old adult group it showed an excellent sensitivity and specificity as well. The ability of CAT-V total score to discriminate PwADD from PwtD in the old adult group was good, whilst for the oldest-old adults, the CAT-V discriminated PwADD from PwtAD perfectly (Figure 2). Cutoff scores for CAT-V in detail are also presented in Table 4.

## 5. Discussion

The main aim of the current study was to identify whether voting capacity in its different aspects can be predicted by the cognitive status in older adult populations experiencing different rates of cognitive decline, as measured by the understanding of nature and effect of voting, of making a choice, of reasoning about voting choices, of comparative reasoning, and of appreciating the reality of the voting situation, which constitute standard decision-making abilities related to voting [14]. A secondary aim was to investigate whether PwtD are capable of voting in comparison to PwADD, according to the total CAT-V score.

The current findings confirm our first hypothesis, since the total MMSE score is positively associated with the CAT-V, meaning that increased CAT-V scores are a prerequisite for increased cognitive function. However, some CAT-V items/categories are weighted more than others. In more detail, the results showed that both the nature and results/effects of voting require an MMSE score > 19.50, meaning that these processes have low cognitive demands, as older adults with mild to moderate ADD can correctly respond. The next CAT-V items, voting choice and appreciation, have increased cognitive requirements, since an MMSE cutoff of 22.50 and 23.50, respectively, implies that only older adults with mild ADD can understand them. Finally, the most cognitively demanding CAT-V tasks were those requiring comparative and consequential reasoning, with cutoff scores of 25.50 and 27.50, respectively, indicating that only cognitively healthy older adults are able to vote when all aspects of voting are concerned. In other words, the aforementioned findings support that comparative and consequential reasoning cannot be applied in cognitively impaired older adults, since they both require increased general cognitive function, at least as measured by the MMSE. Comparative, as well as consequential, reasoning are closely related to executive functioning, which encompasses a broad range of cognitive abilities involved in goal-oriented actions. Specifically, comparative reasoning is the fundamental skill of employing comparisons to reach a conclusion, whereas consequential reasoning has to do with evaluating the consequences of an action or understanding their outcome effects. Therefore, both types of reasoning are mainly involved in the election process, because inability to (a) compare between alternative candidates and (b) evaluate the consequences of election preferences could constitute the vote invalid. Additionally, despite not being able to localize executive function in specific brain areas, there is strong evidence that the prefrontal cortex (which is primarily impaired in ADD), constitutes their neural substrate. Hence, the deficits in the aforementioned functions constitute symptoms of ADD according to the DSM-5. To sum up, given that voting is a multifactorial process with various cognitive demands, it seems that not only people with AD but even people with MCI due to AD are not able to adequately respond to the entirety of them, and therefore their participation in elections is questionable.

Our findings are in line with the study of Irastorza et al. (2007; 2011) [14,38], who found that the nature as well as the effect of voting, along with appreciation, do not require increased cognitive function, in contrast to voting choice and consequential reasoning. Moreover, they also stress that the MMSE total score is positively correlated with the total CAT-V score. Additionally, Appelbaum et al. (2005) [15] found that patients with very mild to mild ADD retained a general ability to vote, whereas those with severe ADD did not. According to their findings, the most controversial group of their study, concerning their voting capacity, was the one with moderate ADD. The results of the current study agree with those of Appelbaum et al. (2005) [15], since we found that increased CAT-V scoring is positively associated with increased MMSE scores and therefore prerequisite increased cognitive demands. However, our findings indicate that only healthy older adults (with MMSE score > 27) are able to understand all aspects of voting. Finally, Raad et al. (2009) [39] found no correlation between MMSE total score and CAT-V; nevertheless, the sample of their study included patients with mental illnesses, and therefore it can be inferred that voting capacity is severely affected by cognitive deterioration due to major neurocognitive disorders, rather than other types of mental disorders.

Moving on to the six items of CAT-V, Tiraboschi et al. (2011) [25] support that expressing a choice was the least affected of the decision-making abilities; however, taking into consideration the small number of participants recruited in their study, this matter still remains unclear. Moreover, Tiraboschi et al. (2011) found a minority of older adults with dementia in a mild stage retained their voting capacity (CAT-V total score) (MMSE ≥ 20), whereas the one third of those with moderate dementia achieved the maximum score in the six CAT-V items [25]. Furthermore, according to their findings, the understanding and choice items were feasible for the 58% of those with MMSE ≤ 20. A possible explanation of this discrepancy between our study with that of Tiraboschi et al. (2011) could be attributed to the different data samples, as well as the fact that Tiraboschi et al. (2011) did not recruit older adults with severe dementia, and therefore, the range of participants’ cognitive decline was quite small [25]. Therefore, only older adults with PwtD presented with a full capacity to vote, as expressed by the sum of the six CAT-V items in the current study, a conclusion also reached by most of the aforementioned studies. Moreover, older adults with severe ADD could be excluded from their voting rights, whereas data about those with moderate ADD are still questionable, which is also supported by Irastorza et al. (2007) [31]. Finally, it is worth mentioning that voting capacity constitutes a multivariable process which includes tasks with different cognitive demands. Therefore, clinicians’ assessments should focus on impaired decision making, since this seems to influence voting capacity and competency in a multidimensional way.

The results of our study also stress that increased age, along with lower MMSE total scores, are associated with lower total voting function, at least as measured by CAT-V. However, due to the small number of participants who belonged in the age group of 86–95, this conclusion should be approached with some caution. Furthermore, it is noteworthy that neither gender nor education levels affected voting, meaning that being able to participate in elections requires cognitive demands that are independent from the participants’ educational level or gender. To conclude, to our knowledge, no previous studies have measured the possible role of demographics in voting capacity, constituting a need for further research on this field.

In accordance with what has been initially hypothesized (Hypothesis 2), the higher the total score of CAT-V, the higher the MMSE score, discriminating older adults with cognitive impairment from the cognitively intact older adult population. As obvious from Table 3, the mean scores of the total voting capacity measured by the CAT-V sum score were significantly increased in PwtD in comparison to those with ADD. Given that increased CAT-V scores indicate increased voting capacity, only PwtD maintain their full ability to vote. Hence, without taking into account the CAT-V sub scores, the total voting capacity is fully supported by PwtD, despite the different cognitive demands included in the six CAT-V sub-tests.

According to the ROC curve analyses, the CAT-V total score has better discriminant validity in the middle-aged as well as the very old adult population in comparison to the other two age groups. In more detail, the CAT-V cutoff score ≥ 8 (out of 12) indicates a middle-aged adult who is able to provide intact voting competence, whereas the cutoff score ≥ 9.5 indicates a very old older adult who also maintains their full voting capacity. The CAT-V total cutoff scores from the other two age categories provide satisfactory AUC values; however, their specificity and sensitivity are not adequate. In detail, the specificity score of the 10.5 cutoff was almost perfect in the 66–75 aged group, but its sensitivity score was relatively low. On the contrary, the total CAT-V cutoff score of 7.5 in the 76–85 age group had a very satisfactory sensitivity index, but nevertheless its specificity was rather low. To the best of our knowledge, no previous study has used the CAT-V to differentiate PwADD from PwtD, and therefore future research should shed more light on this issue, taking into account other categories of cognitive decline such as MCI and SCD due to AD pathology.

To conclude, due to the limited studies about voting capacity in older adults with mild and/or moderate cognitive impairment, the current study aims to offer additional evidence about this crucial issue in the relevant literature. In more detail, the current study focused on determining voting competence, based on (a) understanding the nature and effect of voting, (b) older adults’ ability to choose, (c) reasoning about voting choices, and (d) appreciating the reality of the voting situation. According to our results, screening instruments such as the CAT-V could be administered in older adults when their voting ability was questionable, because MMSE sum score is positively related to CAT-V. Hence, new national strategic plans or law policy makers could use these results and change the prerequisites to vote, so as to obtain more fair voting results, and therefore protect PwADD from possible exploitation concerning their participation in the voting process. On the other hand, CAT-V was found to classify older adults with PwADD and PwtD. The most significant evidence of the current study is the differential cutoff scores provided by the six CAT-V items, which highlight that voting capacity constitutes a decision-making process which is a multifaced ability that can be totally supported only by cognitively healthy older adults. The fact that age group, along with MMSE level, are associated with participants’ voting capacity highlights that voting capacity should be evaluated in more detail because it is assumed as a complex ability otherwise deteriorated by age, but not entirely dependent on cognitive impairment. Furthermore, to the best of our knowledge, this is the first study calculating cutoff scores for each of the separate CAT-V variables, and therefore future studies should confirm their accuracy. Another significant point of the current study is that PwADD do not have full voting competence because reasoning ability, either consequential or comparative, is fully accomplished only in PwtD. Therefore, the current study illustrates that voting capacity includes cognitive demands seen in PwtD. Finally, it seems that those with severe ADD may lose their voting capacity, due to lack of their voting competence in almost the majority of voting sub-tests.

## 6. Limitations

It should be stated that the findings of this research should be used with caution due to its possible future use in clinical or law environments. A possible limitation could be the representativeness of our sample. In fact, participants of the current study were recruited from the clinical care settings of the Greek Association of Alzheimer Disease and the Institute of Research in Crete, and therefore they did not belong in the general older adult population. Another limitation of this study is the absence of CAT-V comparison to other screening tools, such as the MoCA test, which has increased psychometric properties compared to MMSE. Moreover, the fact that our sample groups were not matched to demographics, especially age, which was found to affect CAT-V scores, could have an impact on our results. Finally, further research trials about any possible relationship between CAT-V scores with IADL assessment would certainly provide fruitful knowledge on this field, since voting can be assumed to be an activity of independent living.

## 7. Conclusions

The greatest contribution of the current study is that, for the first time in Greece, the ability to vote in people over 50 years of age, with or without cognitive deficits, is investigated. According to our results, only people without dementia are capable of voting, whilst people with Alzheimer’s disease lack full voting competence. It is worth mentioning that several people with Alzheimer’s disease, and therefore with difficulties in voting ability, may be active voters. This means that there are a number of votes that should not been have cast, but they have participated in the final election’s result, possibly affecting the winners of the elections. Finally, the current research provides cutoff scores that can be utilized for defining the voting difficulties with accuracy.

Even though the results of the study are satisfactory, more work in the field of voting capacity is required. Therefore, our future goals are first to define whether people with MCI maintain the cognitive abilities associated with voting capacity intact, or if they present difficulties, and in which parameter of CAT-V, since neurodegeneration may present problems early in the course of AD and therefore in MCI.

Secondly, besides the fact that the CAT-V seems to be a useful tool, it will be interesting to design an enhanced assessment test based on CAT-V. The new test could be designed in order to avoid subjectivity by making the scoring process more quantitative via more standardized questions and answers (e.g., multiple choice), in order to test whether users can select only the answers that are properly substantiated.

## Figures and Tables

**Table 1:** Demographic characteristics of the participants of the study.

Demographic Characteristics	Sample (*n* = 391)
Gender (Male/Female)	*n* = 105/286
Age [M (SD)]	72.04 (8.69)
Age range	51–95
Education in years [M (SD)]	10.98 (4.71)
Age groups	
Μiddle-aged adults	51–65: *n* = 94
Young-old adults	66–75: *n* = 155
Old adults	76–85: *n* = 120
Oldest-old adults	86–95: *n* = 22
Educational level groups (according to years of schooling)	
Low	0–6: *n* = 121
Middle	7–12: *n* = 133
High	>13: *n* = 137

**Table 2:** Frequencies of the values ‘none’ ‘doubtful’, and ‘adequate voting’ of the CAT-V in the total sample.

CAT-V’s Sub-Questions	CAT-V’s Scores		
	No Voting Capacity *n* (%)	Doubtful Voting Capacity *n* (%)	Adequate Voting Capacity *n* (%)
Understands the nature of voting	44 (11.3)	13 (3.3)	334 (85.4)
Understands the results/effects of voting	59 (15.1)	16 (4.1)	316 (80.8)
Choice	63 (16.1)	9 (2.3)	319 (81.6)
Comparative Reasoning	111 (28.4)	39 (10.0)	241 (61.6)
Consequential Reasoning	142 (36.3)	55 (14.1)	194 (49.6)
Appreciation	53 (13.6)	36 (9.2)	302 (77.2)

**Table 3:** Mean (M) and standard deviation (SD) of CAT-V performance in people with Alzheimer’s disease dementia (PwADD) and people without Alzheimer’s disease dementia (PwtD), depending on their age group.

Age Group	CAT-V Score				
	PwADD		PwtD		*p*-Value
Years	M (SD)	*n*	M (SD)	*n*	
51–65	4.77 (1.00)	7	11.11 (0.38)	88	0.000
66–75	6.43 (0.48)	35	10.78 (0.29)	120	0.000
76–85	4.59 (0.55)	51	10.08 (0.32)	64	0.000
86–95	1.61 (0.81)	11	12.00 (1.22)	4	0.000

Abbreviations: *p*: statistical significance.

**Table 4:** Diagnostic CAT-V classification between people without Alzheimer’s disease dementia (PwtD), and people with Alzheimer’s disease dementia (PwADD).

	CAT-V Cutoff	AUC	Sensitivity %	Specificity %	95% CI	*p*-Value
Age Categories	PwtD vs. PwADD					
51–65 years	8.00	0.974	93.2	100	0.943–1.000	<0.001
66–75 years	10.5	0.920	73.3.	97.1	0.877–0.962	<0.001
76–85 years	7.5	0.834	92.2	64.7	0.758–0.911	<0.001
86–95 years	9.5	1.000	100	100	1.000–1.000	0.004

Abbreviations: *p*: significant difference; AUC: Area Under the Curve; CI: Confidence interval.

## Data Availability

No data are reported.
